# Supplementary material for: Evaluating water quality impacts on visitation to coastal recreation areas using data derived from cell phone locations
Source: PLoS One. 2022 Apr 27;17(4):e0263649. doi: 10.1371/journal.pone.0263649 (PMC9045601; doi:10.1371/journal.pone.0263649)
Supplement: S1 Appendix — (DOCX) [file pone.0263649.s001.docx]

**S1 Appendix: The Clean Water Act, beach monitoring, and what happens when a sample tests positive for contamination**

Section 303(d) of the Clean Water Act (CWA) requires states to identify and list their impaired water bodies. Through a cohort of EPA programs (such as establishing Total Maximum Daily Loads (TMDLs)), states are provided a general framework through which they can work towards recovering the quality of a body of water within their jurisdiction. In practicality, the governance of water quality, and therefore the process of managing water quality impairment, varies greatly between states, water types, and localities. The majority of water quality sampling in New England falls under the jurisdiction of state and county boards of health. In Massachusetts, bathing beaches (saltwater beaches) are monitored during the bathing season (typically from Memorial Day to Labor Day), though exact sampling dates vary by location. The majority of marine beaches are tested at least once a week, depending on a beach’s closure history. The methodology and protocol for beach monitoring in Massachusetts is generally representative of beach monitoring across New England. Testing most public beaches from Memorial Day to Labor Day is typical although specific intervals between tests, protocol, and thresholds for contamination classification do differ geographically.

While the protocol for sampling water quality at bathing beaches does vary at state and local levels, the specific methods for testing water quality are generally consistent across the New England region. Once a water sample (or multiple samples if the beach is large) is retrieved (normally in waist-deep water) the normal protocol requires that sample to be processed by a water quality testing lab (often third-party contractors) within 6 hours. The lab usually processes the sample and generates results in a 24-hour time period. In the case that the sample contains a concentration of pathogens which exceeds federal and state thresholds, then a “re-test” is immediately ordered for that specific beach. If the subsequent also exceeds thresholds, that beach is “closed” to swimming for the subsequent 24-hour period. In addition to posting advisory and closure signs at the access site of the contaminated beaches, states have made concerted efforts to make data readily available to the public through websites (such as the Massachusetts Beach Water Quality Locator or the Maine Healthy Beaches (MHB) Program), outreach programs (such as the MHB program), and education of the public. Connecticut’s department of public health lists public notification procedures for each municipal agency, which include postings at beaches and online, in the newspaper, and via radio and tv.

The specific mechanism that closes a beach to swimming or bathing is not precise and varies by state. Certain states (like Maine, New Hampshire, and Rhode Island) post “advisories” that are suggestions to beach-goers to avoid contact with water. These states’ laws contain provisions which keep beach postings the responsibility of local jurisdictions. Other states (such as Massachusetts) have it written into law that swimming or bathing are prohibited when water quality does not meet requirements. Furthermore, in states like New Hampshire and Maine, local beach managers and boards of health retain the right to keep a beach open or state a beach is closed using their own discretion. Across New England, beach closures and swimming advisories do not prohibit the use of a beach for land-based activities (walking, sports, etc).
