# Supplementary material for: Evaluating water quality impacts on visitation to coastal recreation areas using data derived from cell phone locations
Source: PLoS One. 2022 Apr 27;17(4):e0263649. doi: 10.1371/journal.pone.0263649 (PMC9045601; doi:10.1371/journal.pone.0263649)
Supplement: S1 Table — (DOCX) [file pone.0263649.s002.docx]

**S1 Table. Monitoring records by state for the 2017 bathing season**

| **2017 Summer Bathing Season (Memorial Day through Labor Day)** | **Number of monitored locations** | **Number of locations with closure or advisory events** | **Marine beach closure (or advisory) events** | **Marine beach closure (or advisory) days** | **Number of samples collected** | **% samples above threshold** |
| --- | --- | --- | --- | --- | --- | --- |
| **Maine** | 109 | 16 | 23 | 36 | 1,296 | 2.7% |
| **New Hampshire** | 16 | 7 | 9 | 21 | 842 | *n/a* |
| **Massachusetts** | 583 | 169 | 330 | 429 | 7,844 | 4.1% |
| ***Cape Cod*** | 259 | 8 | 8 | 20 | 3,163 | 2.4% |
| **Rhode Island** | 69 | 27 | 28 | 73 | 1,586* | *n/a* |
| **Connecticut** | 73 | 32 | 60 | 154 | 1,981 | 5.3% |
| TOTALS | **850** | **251** | **450** | **713** | **13,549** | ***N/A*** |

Table S1
