# Supplementary material for: Evaluating water quality impacts on visitation to coastal recreation areas using data derived from cell phone locations
Source: PLoS One. 2022 Apr 27;17(4):e0263649. doi: 10.1371/journal.pone.0263649 (PMC9045601; doi:10.1371/journal.pone.0263649)
Supplement: S2 Table — (DOCX) [file pone.0263649.s003.docx]

**S2 Table. Comprehensive results for multiple subsets of the coastal recreation areas.**

| *Dependent variable:* Log of Prediction | | | | | | | | | | |  |
| --- | --- | --- | --- | --- | --- | --- | --- | --- | --- | --- | --- |
|  |  | | | | | | | | | | |
|  | All | All w/ lags | Cape only | Big cape | Off cape | Saltwater | Fresh water | Monitored | Dense (3^rd^ q) | Most closed (3^rd^ q) | Most closed 2 (>=2) |
|  | | | | | | | | | | | |
| **temp** | 0.043^***^ | 0.046^***^ | 0.044^***^ | 0.056^***^ | 0.052^***^ | 0.048^***^ | 0.034^***^ | 0.053^***^ | 0.049^***^ | 0.047^***^ | 0.044^***^ |
|  | (0.0003) | (0.0004) | (0.0005) | (0.001) | (0.001) | (0.0005) | (0.001) | (0.001) | (0.001) | (0.001) | (0.001) |
|  |  |  |  |  |  |  |  |  |  |  |  |
| **wind** | 0.012^***^ | 0.006^***^ | 0.006^***^ | -0.006 | 0.004 | 0.004^**^ | 0.016^***^ | 0.0003 | -0.005 | 0.003 | 0.003 |
|  | (0.001) | (0.002) | (0.002) | (0.004) | (0.004) | (0.002) | (0.004) | (0.002) | (0.003) | (0.005) | (0.005) |
|  |  |  |  |  |  |  |  |  |  |  |  |
| **precip** | -0.228^***^ | -0.240^***^ | -0.233^***^ | -0.308^***^ | -0.267^***^ | -0.252^***^ | -0.165^***^ | -0.276^***^ | -0.226^***^ | -0.219^***^ | -0.201^***^ |
|  | (0.004) | (0.004) | (0.004) | (0.009) | (0.009) | (0.004) | (0.009) | (0.006) | (0.008) | (0.010) | (0.012) |
|  |  |  |  |  |  |  |  |  |  |  |  |
| **jun** | -0.186^***^ | -0.204^***^ | -0.217^***^ | -0.274^***^ | -0.165^***^ | -0.215^***^ | -0.141^***^ | -0.222^***^ | -0.161^***^ | -0.164^***^ | -0.136^***^ |
|  | (0.008) | (0.009) | (0.010) | (0.020) | (0.020) | (0.010) | (0.022) | (0.013) | (0.018) | (0.023) | (0.028) |
|  |  |  |  |  |  |  |  |  |  |  |  |
| **jul** | 0.053^***^ | -0.127^***^ | -0.140^***^ | -0.145^***^ | -0.089^***^ | -0.119^***^ | -0.160^***^ | -0.115^***^ | -0.099^***^ | -0.100^***^ | -0.061^*^ |
|  | (0.008) | (0.010) | (0.011) | (0.023) | (0.023) | (0.011) | (0.023) | (0.014) | (0.020) | (0.027) | (0.032) |
|  |  |  |  |  |  |  |  |  |  |  |  |
| **aug** | -0.071^***^ | -0.218^***^ | -0.226^***^ | -0.238^***^ | -0.199^***^ | -0.220^***^ | -0.191^***^ | -0.225^***^ | -0.222^***^ | -0.207^***^ | -0.166^***^ |
|  | (0.008) | (0.010) | (0.011) | (0.023) | (0.022) | (0.011) | (0.023) | (0.014) | (0.020) | (0.026) | (0.032) |
|  |  |  |  |  |  |  |  |  |  |  |  |
| **tue** | -0.047^***^ | -0.035^***^ | -0.034^***^ | -0.062^***^ | -0.042^**^ | -0.037^***^ | -0.037^**^ | -0.058^***^ | -0.045^***^ | 0.006 | 0.003 |
|  | (0.007) | (0.008) | (0.009) | (0.018) | (0.018) | (0.009) | (0.018) | (0.011) | (0.016) | (0.021) | (0.025) |
|  |  |  |  |  |  |  |  |  |  |  |  |
| **wed** | 0.039^***^ | 0.083^***^ | 0.074^***^ | 0.170^***^ | 0.122^***^ | 0.091^***^ | 0.044^**^ | 0.112^***^ | 0.024 | 0.111^***^ | 0.085^***^ |
|  | (0.007) | (0.009) | (0.010) | (0.020) | (0.020) | (0.009) | (0.019) | (0.012) | (0.017) | (0.023) | (0.027) |
|  |  |  |  |  |  |  |  |  |  |  |  |
| **thu** | 0.043^***^ | 0.035^***^ | 0.042^***^ | 0.028^*^ | 0.021 | 0.026^***^ | 0.101^***^ | 0.006 | 0.001 | 0.029 | 0.014 |
|  | (0.006) | (0.008) | (0.009) | (0.017) | (0.017) | (0.008) | (0.017) | (0.011) | (0.015) | (0.020) | (0.024) |
|  |  |  |  |  |  |  |  |  |  |  |  |
| **fri** | 0.034^***^ | 0.104^***^ | 0.095^***^ | 0.103^***^ | 0.130^***^ | 0.116^***^ | 0.007 | 0.113^***^ | 0.126^***^ | 0.121^***^ | 0.107^***^ |
|  | (0.007) | (0.008) | (0.009) | (0.018) | (0.018) | (0.009) | (0.018) | (0.011) | (0.016) | (0.021) | (0.026) |
|  |  |  |  |  |  |  |  |  |  |  |  |
| **sat** | 0.178^***^ | 0.265^***^ | 0.263^***^ | 0.273^***^ | 0.270^***^ | 0.280^***^ | 0.154^***^ | 0.273^***^ | 0.280^***^ | 0.275^***^ | 0.260^***^ |
|  | (0.007) | (0.009) | (0.010) | (0.020) | (0.020) | (0.009) | (0.020) | (0.012) | (0.017) | (0.023) | (0.027) |
|  |  |  |  |  |  |  |  |  |  |  |  |
| **sun** | 0.324^***^ | 0.347^***^ | 0.316^***^ | 0.415^***^ | 0.457^***^ | 0.368^***^ | 0.210^***^ | 0.414^***^ | 0.327^***^ | 0.431^***^ | 0.375^***^ |
|  | (0.006) | (0.008) | (0.009) | (0.018) | (0.018) | (0.009) | (0.018) | (0.011) | (0.015) | (0.021) | (0.025) |
|  |  |  |  |  |  |  |  |  |  |  |  |
| **closed** | **-0.001** | **-0.003** | **-0.164^**^** | **-0.253^**^** | **0.021** | **-0.003** |  | **0.0004** | **-0.011** | **0.004** | **0.002** |
|  | **(0.023)** | **(0.024)** | **(0.074)** | **(0.100)** | **(0.027)** | **(0.024)** |  | **(0.025)** | **(0.033)** | **(0.024)** | **(0.026)** |
|  |  |  |  |  |  |  |  |  |  |  |  |
| **lag_log_1** |  | 0.203^***^ | 0.211^***^ | 0.163^***^ | 0.174^***^ | 0.195^***^ | 0.239^***^ | 0.176^***^ | 0.213^***^ | 0.198^***^ | 0.204^***^ |
|  |  | (0.004) | (0.005) | (0.009) | (0.009) | (0.005) | (0.013) | (0.006) | (0.008) | (0.011) | (0.014) |
|  |  |  |  |  |  |  |  |  |  |  |  |
| **lag_log_2** |  | 0.015^***^ | 0.017^***^ | 0.017^*^ | 0.012 | 0.014^***^ | 0.028^**^ | 0.014^**^ | 0.041^***^ | 0.030^**^ | 0.042^***^ |
|  |  | (0.005) | (0.005) | (0.009) | (0.009) | (0.005) | (0.013) | (0.006) | (0.009) | (0.012) | (0.015) |
|  |  |  |  |  |  |  |  |  |  |  |  |
| **lag_log_3** |  | -0.005 | -0.003 | -0.029^***^ | -0.007 | -0.006 | 0.019 | -0.008 | 0.004 | 0.008 | 0.007 |
|  |  | (0.005) | (0.005) | (0.009) | (0.009) | (0.005) | (0.013) | (0.006) | (0.008) | (0.012) | (0.015) |
|  |  |  |  |  |  |  |  |  |  |  |  |
| **lag_log_4** |  | 0.073^***^ | 0.078^***^ | 0.081^***^ | 0.054^***^ | 0.073^***^ | 0.057^***^ | 0.071^***^ | 0.080^***^ | 0.054^***^ | 0.045^***^ |
|  |  | (0.005) | (0.005) | (0.009) | (0.009) | (0.005) | (0.013) | (0.006) | (0.008) | (0.012) | (0.015) |
|  |  |  |  |  |  |  |  |  |  |  |  |
| **lag_log_5** |  | 0.012^***^ | 0.019^***^ | 0.030^***^ | -0.001 | 0.009^*^ | 0.068^***^ | 0.016^***^ | 0.004 | 0.020^*^ | 0.016 |
|  |  | (0.005) | (0.005) | (0.009) | (0.009) | (0.005) | (0.013) | (0.006) | (0.009) | (0.012) | (0.015) |
|  |  |  |  |  |  |  |  |  |  |  |  |
| **lag_log_6** |  | 0.021^***^ | 0.022^***^ | 0.030^***^ | 0.026^***^ | 0.022^***^ | -0.007 | 0.027^***^ | 0.024^***^ | 0.033^***^ | 0.024 |
|  |  | (0.005) | (0.005) | (0.009) | (0.009) | (0.005) | (0.013) | (0.006) | (0.008) | (0.012) | (0.015) |
|  |  |  |  |  |  |  |  |  |  |  |  |
| **lag_log_7** |  | 0.098^***^ | 0.098^***^ | 0.057^***^ | 0.084^***^ | 0.091^***^ | 0.121^***^ | 0.075^***^ | 0.124^***^ | 0.090^***^ | 0.101^***^ |
|  |  | (0.005) | (0.005) | (0.009) | (0.009) | (0.005) | (0.013) | (0.006) | (0.008) | (0.012) | (0.015) |
|  |  |  |  |  |  |  |  |  |  |  |  |
| **lag_log_8** |  | -0.026^***^ | -0.025^***^ | -0.019^*^ | -0.020^**^ | -0.027^***^ | -0.007 | -0.022^***^ | -0.029^***^ | -0.0003 | -0.013 |
|  |  | (0.005) | (0.005) | (0.010) | (0.010) | (0.005) | (0.014) | (0.006) | (0.009) | (0.012) | (0.015) |
|  |  |  |  |  |  |  |  |  |  |  |  |
| **lag_log_9** |  | 0.024^***^ | 0.025^***^ | 0.039^***^ | 0.024^**^ | 0.025^***^ | 0.032^**^ | 0.029^***^ | 0.025^***^ | 0.014 | 0.014 |
|  |  | (0.005) | (0.005) | (0.009) | (0.009) | (0.005) | (0.013) | (0.006) | (0.009) | (0.012) | (0.015) |
|  |  |  |  |  |  |  |  |  |  |  |  |
| **lag_log_10** |  | 0.014^***^ | 0.013^**^ | -0.007 | 0.011 | 0.010^**^ | 0.026^**^ | 0.007 | 0.038^***^ | 0.008 | 0.027^*^ |
|  |  | (0.004) | (0.005) | (0.009) | (0.009) | (0.005) | (0.013) | (0.006) | (0.008) | (0.011) | (0.014) |
|  |  |  |  |  |  |  |  |  |  |  |  |
|  | | | | | | | | | | | |
| **Observations** | 51,511 | 38,447 | 29,141 | 8,565 | 9,217 | 33,799 | 4,648 | 22,287 | 11,416 | 5,883 | 3,859 |
| **R^2^** | **0.472** | **0.491** | **0.486** | **0.545** | **0.517** | **0.501** | **0.449** | **0.524** | **0.509** | **0.506** | **0.496** |
| **Adjusted R^2^** | 0.466 | 0.483 | 0.478 | 0.538 | 0.510 | 0.494 | 0.436 | 0.517 | 0.503 | 0.498 | 0.487 |
| **F Statistic** | 3,498.782^***^ (df = 13; 50921) | 1,587.487^***^ (df = 23; 37894) | 1,179.208^***^ (df = 23; 28701) | 439.217^***^ (df = 23; 8437) | 422.490^***^ (df = 23; 9082) | 1,452.814^***^ (df = 23; 33337) | 168.065^***^ (df = 22; 4535) | 1,050.998^***^ (df = 23; 21983) | 508.635^***^ (df = 23; 11264) | 257.602^***^ (df = 23; 5789) | 161.958^***^ (df = 23; 3790) |
|  | | | | | | | | | | | |
| *Note:* | ^*^p<0.1; ^**^p<0.05; ^***^p<0.01 | | | | | | | | | | |
