# Supplementary figures and images for: Evaluating water quality impacts on visitation to coastal recreation areas using data derived from cell phone locations
Source: PLoS One. 2022 Apr 27;17(4):e0263649. doi: 10.1371/journal.pone.0263649 (PMC9045601; doi:10.1371/journal.pone.0263649)

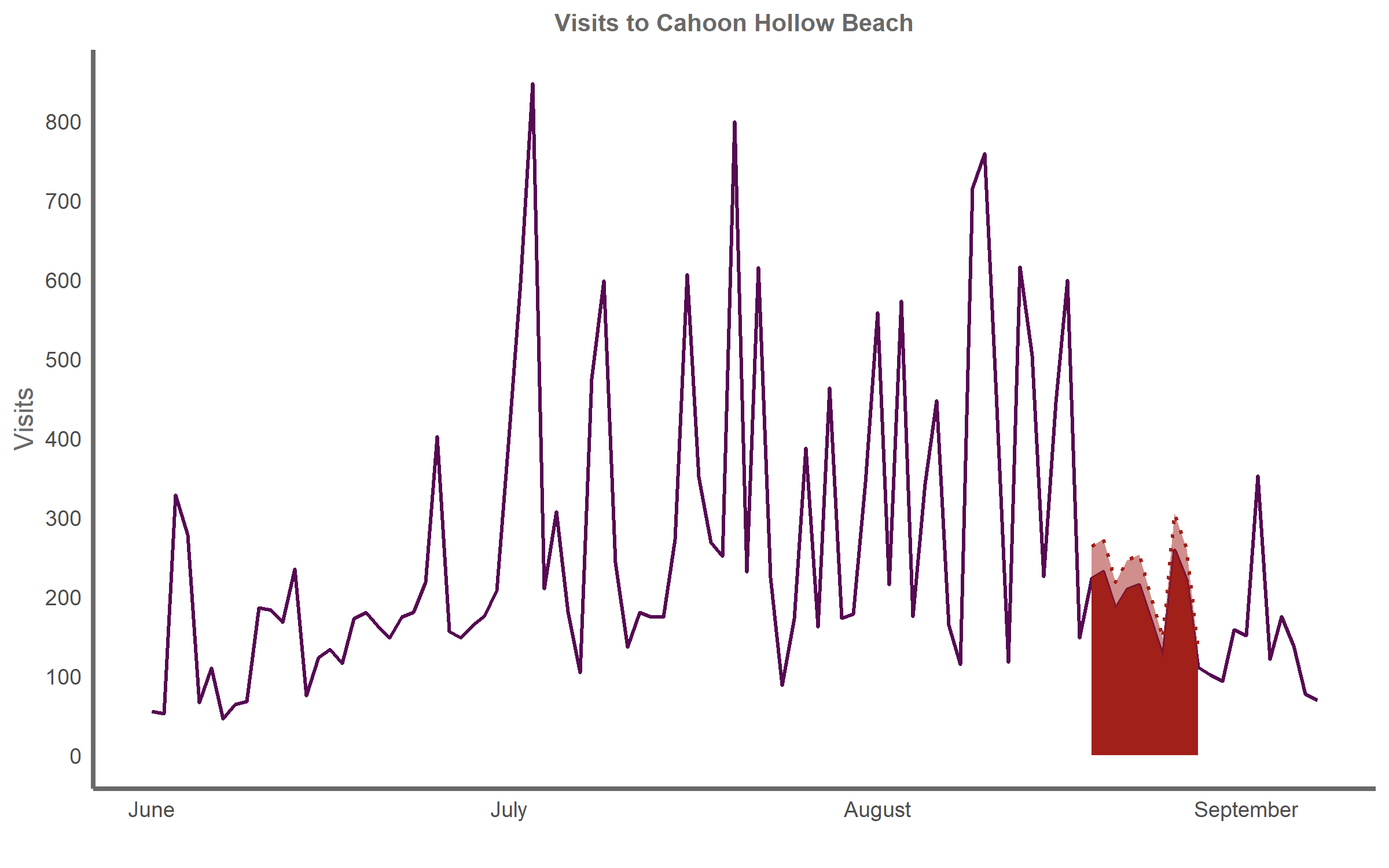

Supplement: S1 Fig — The dark red area beneath the visitation line shows the date range when there was a closure event, and projected visitation (i.e. predicted visitation if there had not been a closure event) is shown in light red below the dotted line. (TIFF) [file pone.0263649.s004.tiff]

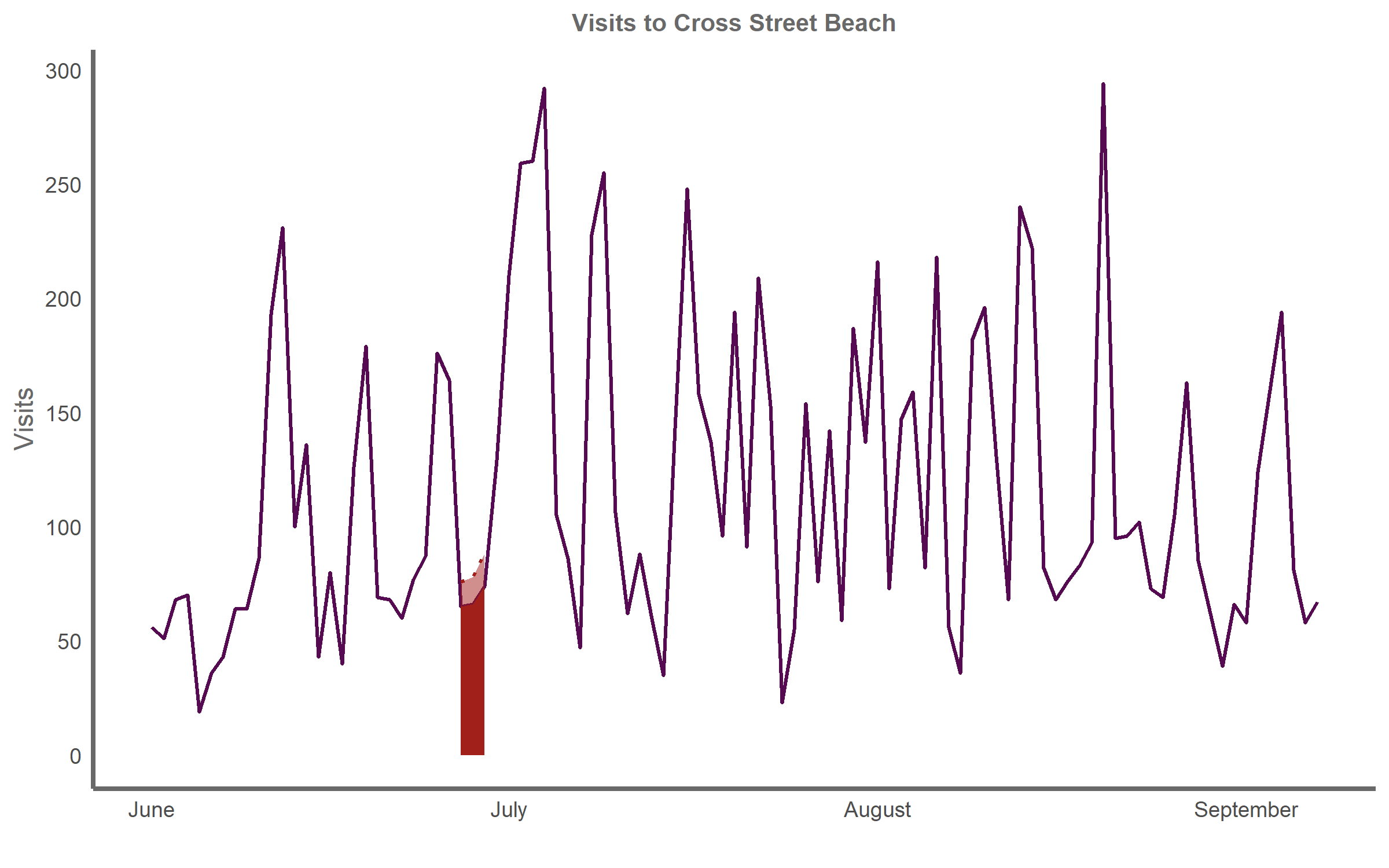

Supplement: S2 Fig — The dark red area beneath the visitation line shows the date range when there was a closure event, and projected visitation (i.e. predicted visitation if there had not been a closure event) is shown in light red below the dotted line. (TIFF) [file pone.0263649.s005.tiff]

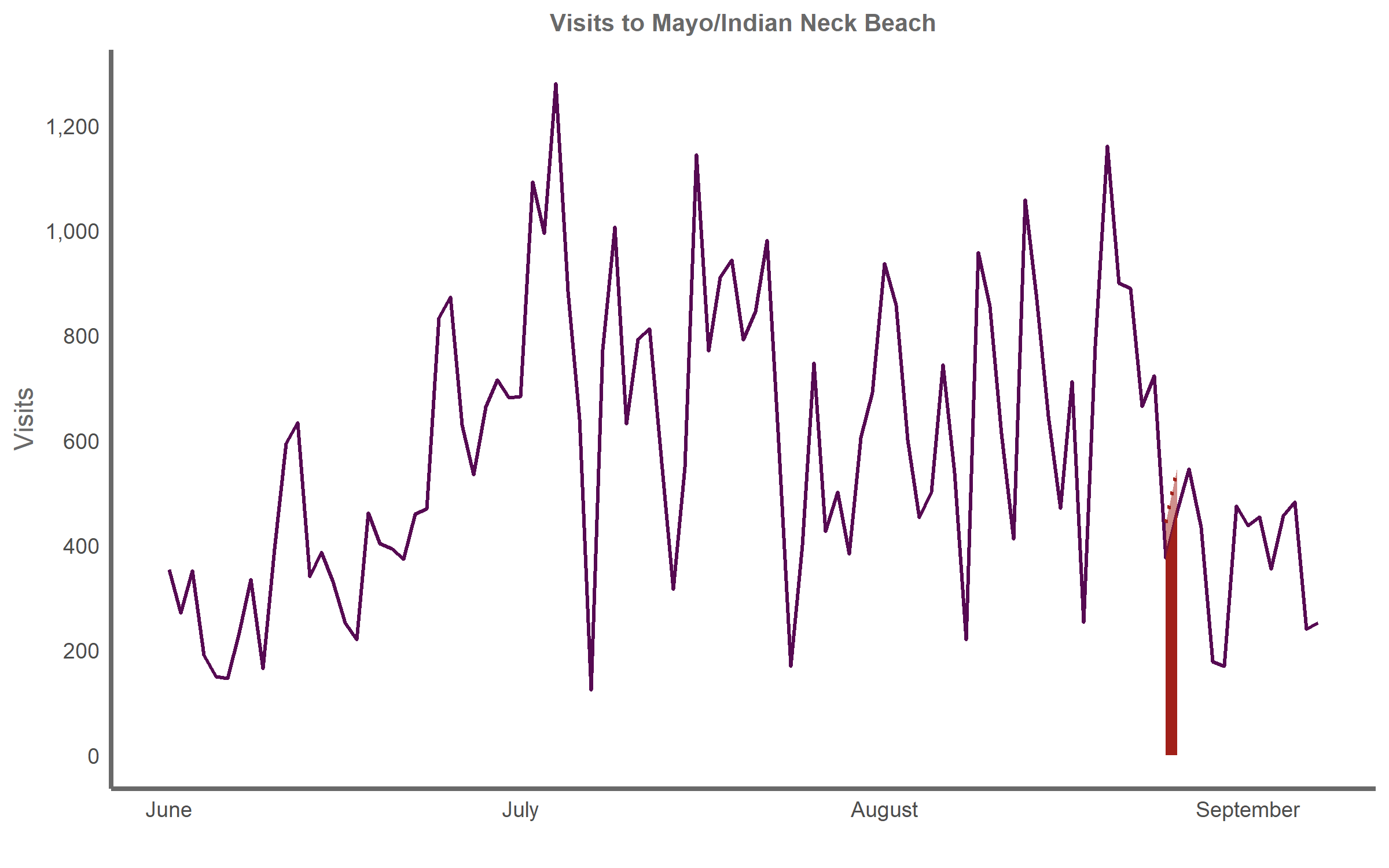

Supplement: S5 Fig — The dark red area beneath the visitation line shows the date range when there was a closure event, and projected visitation (i.e. predicted visitation if there had not been a closure event) is shown in light red. (TIFF) [file pone.0263649.s008.tiff]

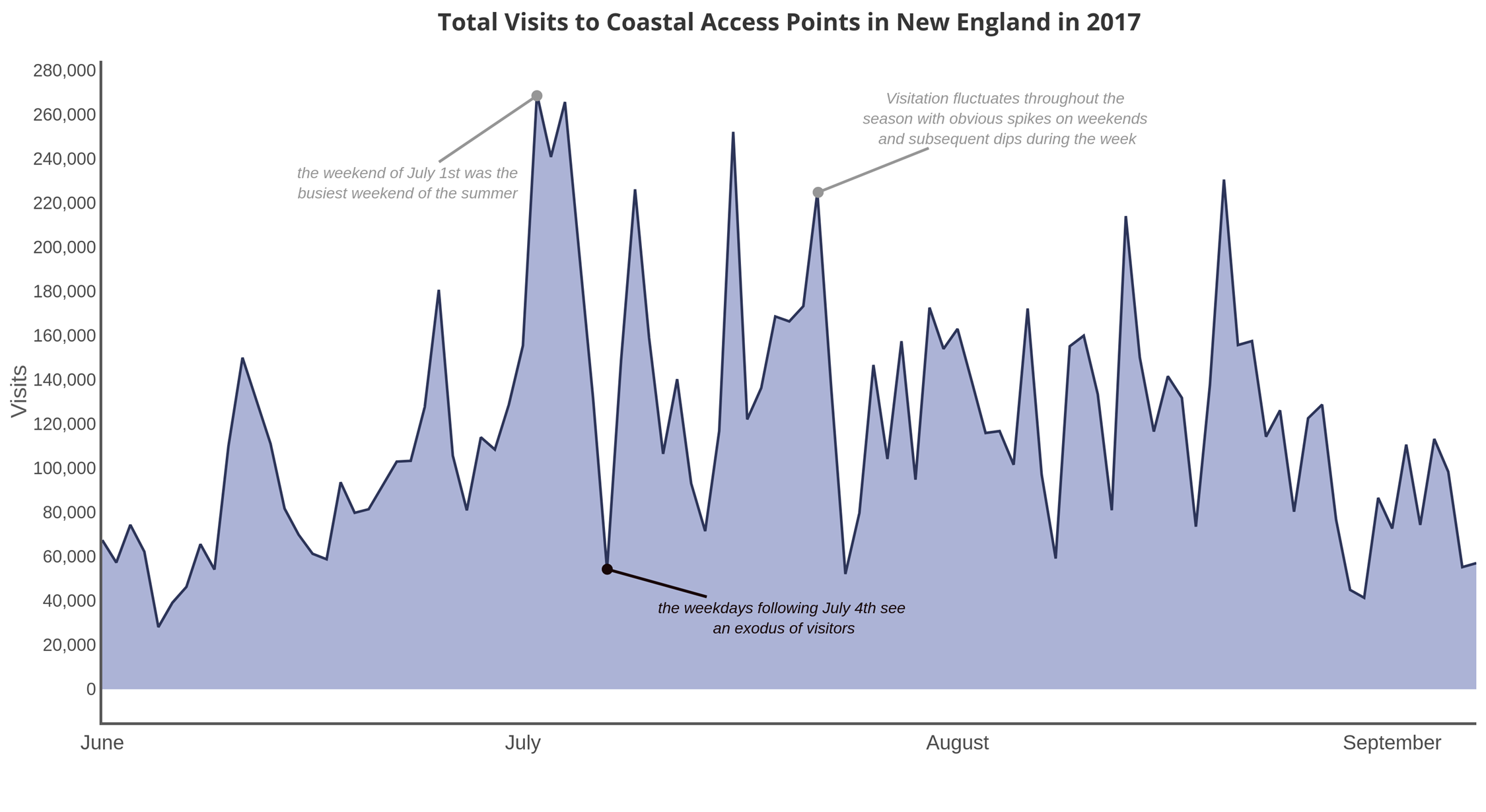

Supplement: S6 Fig — Our sample consists of 465 recreation areas on Cape Cod, and 100 recreation areas across Connecticut, Rhode Island, Massachusetts (off Cape Cod), New Hampshire, and Maine. This figure shows visitation for all 565 recreation areas across the summer season. (TIF) [file pone.0263649.s009.tif]
